# Shortcomings of Vitamin D-Based Model Simulations of Seasonal Influenza

**DOI:** 10.1371/journal.pone.0020743

**Published:** 2011-06-03

**Authors:** Jeffrey Shaman, Christie Y. Jeon, Edward Giovannucci, Marc Lipsitch

**Affiliations:** 1 Department of Environmental Health Sciences, Mailman School of Public Health, Columbia University, New York, New York, United States of America; 2 Center for Infectious Disease Epidemiologic Research, Department of International Center for AIDS Care and Treatment Programs, Mailman School of Public Health, Columbia University, New York, New York, United States of America; 3 Departments of Epidemiology and Nutrition, Harvard School of Public Health, Harvard University, Boston, Massachusetts, United States of America; 4 Center for Communicable Disease Dynamics, Departments of Epidemiology and Immunology and Infectious Diseases, Harvard School of Public Health, Harvard University, Boston, Massachusetts, United States of America; Massey University, New Zealand

## Abstract

Seasonal variation in serum concentration of the vitamin D metabolite 25(OH) vitamin D [25(OH)D], which contributes to host immune function, has been hypothesized to be the underlying source of observed influenza seasonality in temperate regions. The objective of this study was to determine whether observed 25(OH)D levels could be used to simulate observed influenza infection rates. Data of mean and variance in 25(OH)D serum levels by month were obtained from the Health Professionals Follow-up Study and used to parameterize an individual-based model of influenza transmission dynamics in two regions of the United States. Simulations were compared with observed daily influenza excess mortality data. Best-fitting simulations could reproduce the observed seasonal cycle of influenza; however, these best-fit simulations were shown to be highly sensitive to stochastic processes within the model and were unable consistently to reproduce observed seasonal patterns. In this respect the simulations with the vitamin D forced model were inferior to similar modeling efforts using absolute humidity and the school calendar as seasonal forcing variables. These model results indicate it is unlikely that seasonal variations in vitamin D levels principally determine the seasonality of influenza in temperate regions.

## Introduction

Hypotheses attempting to explain the seasonality of epidemic influenza transmission in temperate regions fall into 3 broad categories: 1) seasonal changes in host behavior, mixing patterns and contact rates [1], [2]; 2) seasonal changes in host immune function [3], [4]; and 3) seasonal changes of environmental conditions that affect virus survival and transmissibility [5], [6]. These 3 hypotheses are not mutually exclusive, and influenza transmission dynamics are potentially affected in some fashion by all 3 processes.

Here we explore the second effect, the role that seasonal changes of host immune function may have on influenza infection rates. In particular, we focus on the effect of vitamin D, which is converted from 7-dehydrocholesterol in the skin upon absorption of UVB rays from the sun [3]. The resulting product converts to 25-hydroxy-vitamin D_3_ [25(OH)D] and subsequently to 1,25-dihydroxy-vitamin D_3_, which in combination with vitamin D receptors triggers innate immune responses [7], [8] that may be effective against influenza infection [9], [10], particularly at high levels [11].

An association between vitamin D and likelihood of influenza virus infection was first noted in laboratory experiments with animal models [12]. A study on prevention of industrial absenteeism also found that cod liver oil rich in vitamin D reduced lost time due to respiratory illness [13]. Since those early findings, a number of investigators have hypothesized that the decreased sunlight levels in temperate regions during winter, which decrease vitamin D concentrations and host immune function, increase susceptibility to influenza infection [4], [14]. Further, observational studies and a placebo-controlled trial also found that higher levels of vitamin D or vitamin D supplementation prevented respiratory tract infections [15]–[17]. In this study we explore whether seasonal vitamin D changes are by themselves pronounced enough to modulate influenza infection rates. Specifically, observed seasonal changes in vitamin D levels are here used to modulate the probability of infection of individuals within an agent-based model and determine whether a realistic seasonal cycle of influenza infection rates can be simulated.

## Methods

We compute monthly means and standard deviations of 25(OH)D levels in a sample of individuals enrolled in vitamin D substudies in the Health Professionals Follow-up Study, a prospective investigation of the causes of chronic diseases in male health professionals [18]. We include 722 observations from individuals residing in the Great Lakes region and 701 observations from those in the Northeast region whose blood was drawn between the years 1993–1995 (Table 1).

**Table 1:** 1993–1995 average monthly mean and standard deviation of 25-hydroxy-vitamin D levels for the Great Lakes and Northeast U.S. regions.

	Great Lakes			Northeast		
Month	Number	Mean	Standard Deviation	Number	Mean	Standard Deviation
January	25	24.28	8.39	16	25.44	7.52
February	24	23.05	6.92	23	26.83	9.01
March	47	25.04	9.49	35	23.77	10.32
April	31	24.81	6.35	25	23.59	11.78
May	49	26.60	7.67	57	26.79	9.69
June	94	28.57	8.45	105	27.99	13.85
July	78	32.46	11.34	96	29.51	9.17
August	73	32.14	9.52	85	31.52	9.47
September	156	32.13	10.74	98	31.63	12.62
October	68	28.82	10.70	68	28.65	9.83
November	46	27.94	9.13	60	25.89	9.87
December	31	26.71	8.34	33	24.26	7.53

The Great Lakes includes the states of Illinois, Indiana, Iowa, Michigan, Minnesota, Montana, and Wisconsin. The Northeast includes the states of Connecticut, Delaware, Maine, Maryland, Massachusetts, New Hampshire, New Jersey, New York, Pennsylvania, Rhode Island, West Virginia, Vermont and the District of Columbia.

We use an agent-based version of the perfectly-mixed SIRS model previously described in Shaman et al. [6], but here adapted for forcing with observed 25(OH)D levels, rather than absolute humidity (AH). Briefly, the 25(OH)D level of each individual, or agent, within the model is tracked explicitly. Each individual is ranked and, based on this percentile, assigned a monthly 25(OH)D level using the 1993–1995 average monthly mean and variance of 25(OH)D levels for the region modeled (e.g. the northeastern U.S.); these average monthly 25(OH)D levels were approximately normally distributed. To allow for additional daily variation among individuals, each person was randomly allowed to drift from their prescribed monthly 25(OH)D percentile by ±0.1% per day.

**Figure 1:**
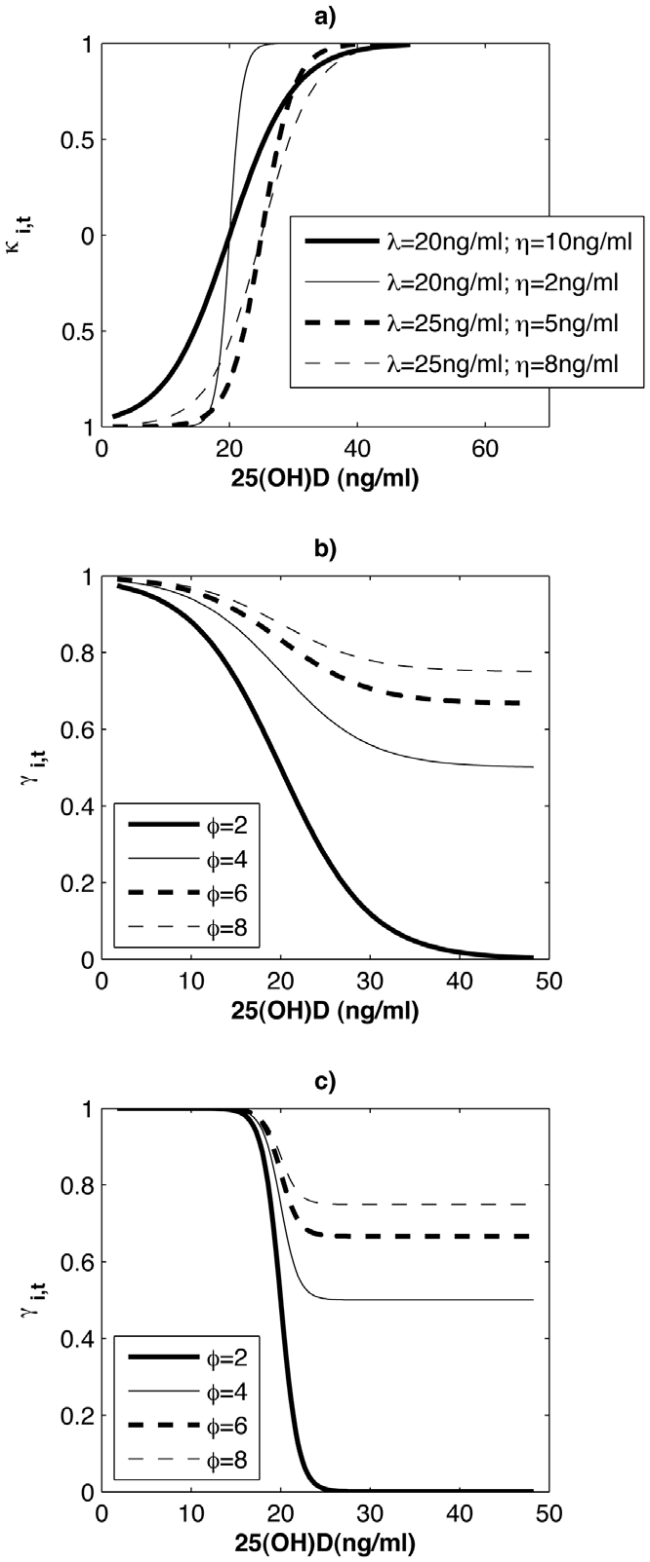
κ_i,t_ and γ_i,t_ plotted as a function of 25(OH)D level for various parameter combinations. a) *κ*_i,t_ plotted for different combinations of *λ* and *η*. b) *γ_i,t_* plotted for *λ* = 20 *ng/ml* and *η* = 10 *ng/ml* and different levels of *ϕ*. c) *γ_i,t_* plotted for *λ* = 20 *ng/ml* and *η* = 2 *ng/ml* and different levels of *ϕ*.



The 25(OH)D level, *V_i,t_,* of individual *i* on day *t* was then transformed into an adjustment of individual likelihood of infection, *γ_i,t_*, via (Figure 1):

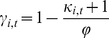
(1)where *κ*_i,t_ is
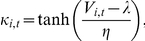



*λ* determines the inflection point of the hyberbolic tangent function, *η* modifies the slope through this inflection, and *ϕ* scales *γ_i,t_* to a value between 0 and 1. By defining the inflection point of the hyperbolic tangent function, *λ* sets the [25(OH)D] level at which *γ_i,t_* changes most precipitously (Figure 1). By modifying the slope through the inflection point, *η* delineates whether the change in *γ_i,t_* as 25(OH)D level varies is gradual or more like a step function.

The daily probability that a susceptible individual is infected, *ν_i,t_* is then scaled by this adjustment:
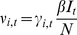
(2)where *β* is the transmission rate constant, *I_t_* is the daily number of infectious people, and *N* is the population size. By construct, persons with higher 25(OH)D levels have smaller *γ_i,t_* and reduced risk of infection. For the population as a whole, the daily mean value of *γ_i,t_* modifies the basic reproduction number, *R*_0_, such that an instantaneous basic reproduction number for the population can be defined as 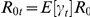. 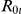
represents the number of secondary cases a primary case would infect if no one were immune and the distribution of *γ_i,t_* were that observed on day *t.* This instantaneous basic reproduction number accounts for changes in the likelihood of infection due to population mean Vitamin D levels; however, this quantity does not account for susceptibility to influenza, i.e. immune status. The actual mean number of secondary cases per case at a given time is the effective reproductive number, which is approximately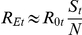 and determines whether total cases are increasing or declining in the population. This relation is approximate because it does not take account of the possible correlation between an individual's vitamin D status and whether or not s/he is in the immune category. The model accounts for such correlations by modeling transmission (and vitamin D status) at the individual level.

The model includes 6 free parameters and was run in ensembles of 3000 simulations. Parameters *λ* and *η* were fixed for all 3000 runs of an ensemble, but varied between ensembles. The remaining 4 parameters—*ϕ*, *R*_0_^*^, the value *R*_0_ would have if *γ_i,t_* equaled one for the entire population, *D*, the mean infectious period, and *L,* the average duration of immunity—were varied among the simulations within an ensemble using a Latin hypercube sampling structure with uniform distribution, as in Shaman et al. [6]. Parameter ranges were: 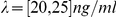; 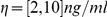; 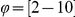; 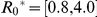; 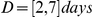 *;* 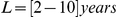. The range of *λ* was assigned to match levels below which parathyroid hormone levels are elevated [3], [19]; the ranges of *R*_0_^*^, *D,* and, *L* match those employed previously for this model [6]; *η* and *ϕ* were varied to consider a wide range of potential responses to higher 25(OH)D levels. *R*_0_^*^ was allowed to drop below critical levels for some simulations, though these few runs fail to sustain continued influenza transmission; more often *R*_0_^*^ was prescribed to be above 2, though daily modulation of *R*_0_^*^ by *γ_i,t_* typically produced instantaneous basic reproduction numbers (*R*_0*t*_) of much lower magnitude within such simulations.

Each simulation used a population of *N = *100,000 persons and was run for 31 years. The model simulates two influenza virus groupings (A-H3N2 and a grouping of the A-H1N1 and B subtypes) without cross immunity. The quality of each simulation was evaluated based on root mean squared (RMS) error with daily observed 1972–2002 excess pneumonia and influenza (P&I) mortality [20], [21]. Simulations for the northeast U.S. region were evaluated with New York state excess P&I mortality. Simulations for the Great Lakes region were evaluated with Illinois state excess P&I mortality.

To test the sensitivity of model outcome to stochastic processes we re-ran the 10 best-fitting parameter combinations for certain ensembles. Each of these parameter combinations was run 100 additional times, each time with different random seeding, to examine the role stochasticity had in producing well-matched simulations. An analogous test was performed in Shaman et al. [6] for simulations forced with either AH or the school calendar.

Additionally, to determine whether the vitamin D-forced simulations were corrupted by the coarse monthly temporal resolution of the data, we interpolated the mean and variance of the monthly vitamin D data to daily values using a cubic spline and used these daily-interpolated values to force the influenza model. Simulations were repeated in this fashion for both the Northeast and Great Lakes regions, and the role of stochasticity was also examined, as described above. For these daily interpolated runs individuals were still ranked but were instead assigned a daily 25(OH)D level based on the daily mean and variance.

Vitamin D simulations were also compared with previously-run simulations forced with either AH or the school calendar for New York state and Illinois; the descriptions and parameterizations of these models can be found in Shaman et al. [6]. In addition, new AH-forced simulations were run for both New York state and Illinois in which the 1972–2002 time series of daily AH conditions for each of these states was replaced with 31-year daily average values. This averaging eliminates year-to-year variability from the AH-forcing and provides a more fair comparison with the vitamin D- and school calendar-forced model runs, which also lack year-to-year variability.

## Results

**Figure 2:**
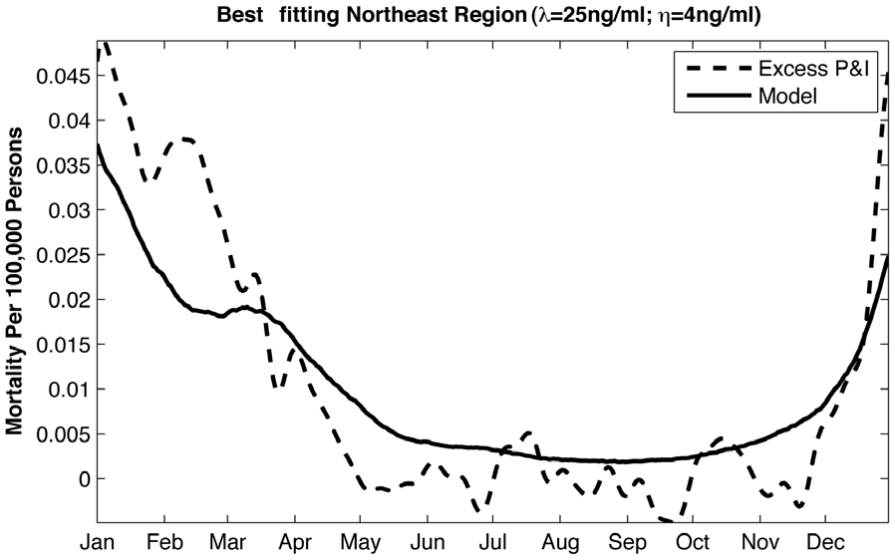
Best-fitting SIRS model simulation for the northeastern U.S. with parameters λ and η fixed at 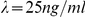 and 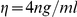. Other parameters are shown in the top line of Table 3. The 31-year simulated mean daily infection number has been scaled to the observed 1972–2002 mean daily excess P&I mortality rate for New York state.

Best-fitting simulations are presented for the Great Lakes region using 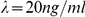 and 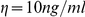 (Table 2) and the northeast U.S. using 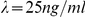 and 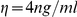 (Table 3). Results with other combinations of *λ* and *η,* as well as with forcing using daily interpolated 25(OH)D levels were comparable (not shown). The quality of these simulations in terms of RMS error and correlation with observed P&I mortality is comparable to simulations with the AH and school calendar forced SIRS model (see Tables S2 and S5 in Shaman et al. [6]). Simulated daily average infection rates capture the seasonal cycle of P&I mortality (Figure 2).

**Table 2:** Parameter combinations for the 10 best-fit simulations for the Great Lakes region as validated with Illinois P&I mortality data.

Rank	RMS Error	Correlation Coefficient (r)	L (years)	D (days)	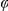	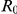 ^*^
1	0.0049	0.93	5.74	4.58	5.11	2.35
2	0.0061	0.87	3.81	2.08	3.33	2.84
3	0.0062	0.84	9.78	2.68	4.92	1.90
4	0.0062	0.86	3.54	3.60	2.85	3.18
5	0.0064	0.86	3.66	3.29	3.67	2.29
6	0.0064	0.88	4.39	6.47	7.13	2.69
7	0.0065	0.83	7.65	2.42	2.79	3.69
8	0.0066	0.82	4.59	2.71	3.30	2.34
9	0.0069	0.82	4.81	6.53	3.15	3.32
10	0.0069	0.91	7.41	5.80	7.71	2.14

3000 simulations were performed at each site with the parameters *L* (mean duration of immunity), *D* (mean infectious period), *ϕ* (vitamin D scaling), and *R*_0_^*^ (the basic reproduction number if *γ_i,t_* = 1) randomly chosen from within specified ranges. Parameters *λ* (inflection point) and *η* (inflection point slope) were fixed at 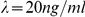 and 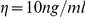. Best-fit simulations were selected based on RMS error after scaling the 31-year mean daily infection number to the 31-year mean observed daily excess P&I mortality rate.

**Table 3:** Parameter combinations for the 10 best-fit simulations for the northeastern U.S. as validated with New York state P&I mortality data.

Rank	RMS Error	Correlation Coefficient(r)	L (years)	D (days)	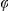	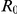 ^*^
1	0.0070	0.94	5.59	5.69	3.83	2.83
2	0.0071	0.94	5.64	5.43	4.99	2.48
3	0.0071	0.90	9.78	5.59	2.43	3.69
4	0.0072	0.88	9.80	3.59	2.55	3.83
5	0.0072	0.90	2.53	6.04	2.86	2.44
6	0.0073	0.89	9.71	3.68	5.54	1.94
7	0.0074	0.89	3.28	4.31	2.13	3.22
8	0.0075	0.90	8.73	3.74	6.79	3.02
9	0.0077	0.91	6.44	5.72	4.24	2.59
10	0.0077	0.91	6.29	3.17	9.27	1.74

3000 simulations were performed at each site with the parameters *L* (mean duration of immunity), *D* (mean infectious period), *ϕ* (vitamin D scaling), and *R*_0_^*^ (the basic reproduction number number if *γ_i,t_* = 1) randomly chosen from within specified ranges. Parameters *λ* (inflection point) and *η* (inflection point slope) were fixed at 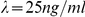 and 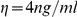. Best-fit simulations were selected based on RMS error after scaling the 31-year mean daily infection number to the 31-year mean observed daily excess P&I mortality rate.

Only a portion of the population seasonally crosses the inflection point for *γ_i,t_* as 25(OH)D levels change (Figure 1b,c) such that only a portion of the population experiences a pronounced modulation of the likelihood of infection. When the slope of *κ*_i,t_ at its inflection is steep (i.e. *η* small) large portions of the population experience little seasonal change in *γ_i,t_*. Still, the portion that is modulated is sufficient at times to phase peak infection during winter and produce a realistic seasonal cycle.

**Figure 3:**
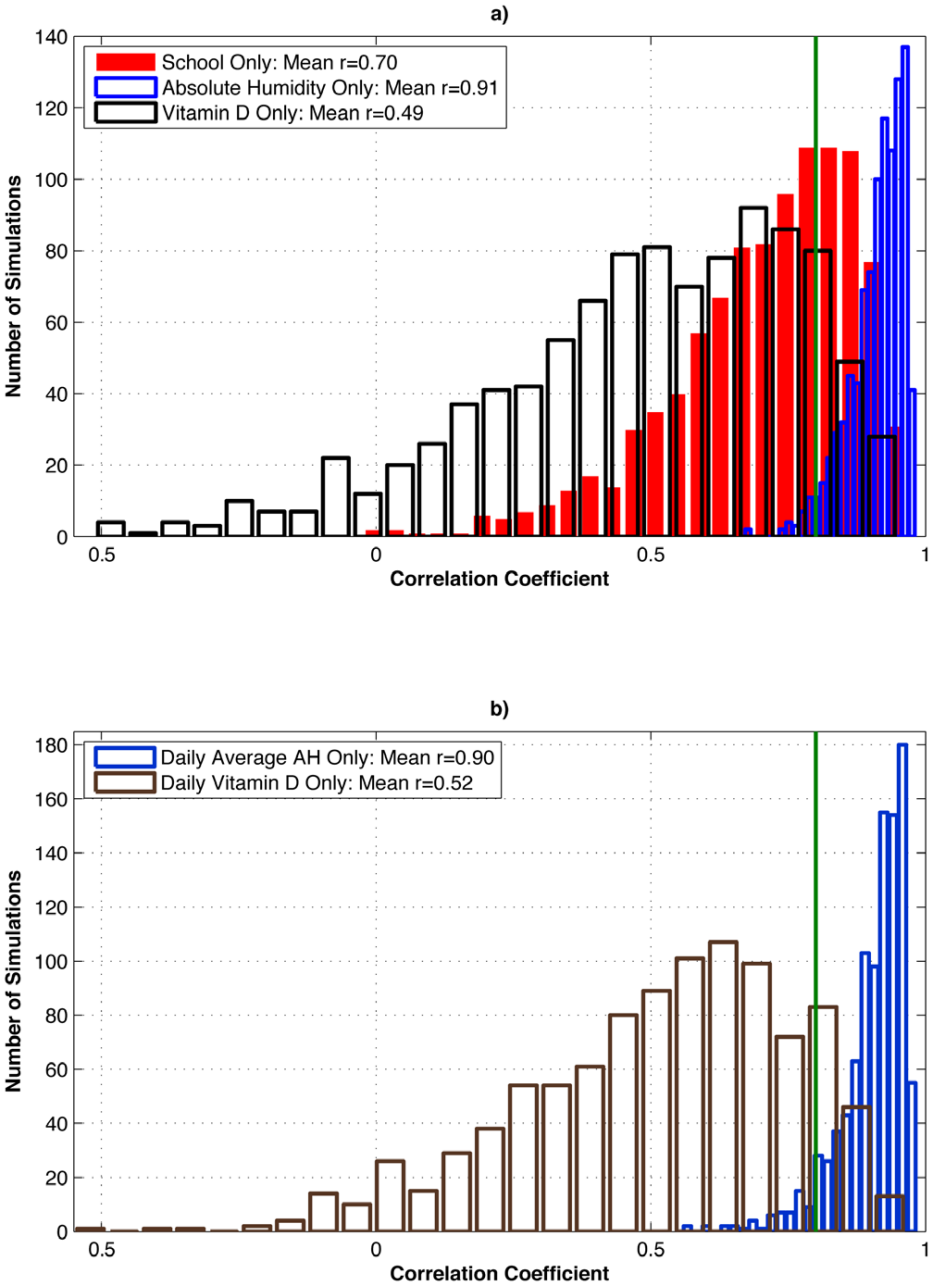
Test of the effect of stochasticity within the SIRS model on well-matched simulations verified with New York state P&I mortality data. a) The 10 best-fit parameter combinations for the SIRS model forced with observed New York school calendar (Shaman et al., 2010: Table S5), observed New York absolute humidity (Shaman et al., 2010: Table S2), and northeastern U.S. vitamin D metabolite levels (Table 3) were each run an additional 100 times, each time with different random seeding. Histograms of correlations with 1972–2002 New York state observed excess P&I mortality are shown. The green line indicates the correlation of an optimally phased sine function with annual periodicity with 1972–2002 New York state observed excess P&I mortality (*r* = 0.80). b) As in a), but for the 10 best-fit simulations using 1972–2002 daily average New York absolute humidity and daily interpolated northeastern U.S. vitamin D metabolite levels.

However, best-fitting model parameter combinations are not consistent among best-fitting runs (Tables 2 and 3), unlike what has been found for simulations forced with observed absolute humidity. Furthermore, some simulations with similar parameter combinations produce anti-correlated seasonal cycles of influenza infection (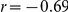). These findings indicate that the quality of fit might be heavily influenced by stochastic events, such that particular parameter combinations could on occasion produce a realistic seasonal cycle, but would not reliably do so. To test this hypothesis, we re-ran the simulations with the top 10 parameter combinations as described in the Methods section. An identical test had previously been performed for SIRS simulations forced with either AH or the school calendar [6] and both these alternate forcing mechanisms prove more resilient to changes in random seeding with AH performing the best (Figure 3).

As a reference, a simple sine function with annual period, if appropriately phased, is highly correlated with the seasonal cycle of observed excess P&I mortality (e.g. *r  = *0.80 for New York state, Figure 3). One might expect that a credible process-based model of the seasonal influenza cycle would consistently improve on this correlation. However, in comparison to school-term forcing and AH forcing, the re-run top parameter sets for 25(OH)D forcing performed considerably worse, as measured by the Pearson correlation between re-run simulations and observations. Specifically, the additional AH forced simulations are much more consistently matched with observations (mean *r* = 0.912; minimum *r* = 0.670; maximum *r* = 0.981) than the additional school calendar forced simulations (mean *r* = 0.704; minimum *r* = −0.024; maximum *r* = 0.962) or the additional vitamin D forced simulations (mean *r* = 0.490; minimum *r*  =  −0.512; maximum *r* = 0.950) (Figure 3a). Both the school and vitamin D models, on average, fall below the correlation level of the naïve sine function model.

A similar test of the effect of stochasticity for the model forced with daily-interpolated vitamin D levels was also not consistently well matched with observations (mean *r* = 0.516; minimum *r*  =  −0.550; maximum *r* = 0.965) (Figure 3b). Conversely, the same test applied to daily-averaged AH-forced simulations was consistently well matched with observations (mean *r* = 0.904; minimum *r* = 0.558; maximum *r* = 0.984). These last two ensembles both use daily forcing without any year-to-year variability, yet the AH-forced model is much more consistently highly correlated with observations than the vitamin D model and on average is better correlated than the naïve sine function model.

## Discussion

Simulation of the seasonal cycle of influenza infection in regions of the U.S. is possible using an SIRS model forced with observed vitamin D levels. However, secondary evidence casts doubt on the validity of this outcome. Parameter combinations that produce a good seasonal cycle of influenza are not similar to one another, and multiple stochastic runs do not reproduce the seasonal cycle reliably, in contrast to similar runs with the other candidate drivers of seasonality, in particular AH.

Any forcing with a strong seasonal cycle will produce an appropriate seasonal cycle of influenza infection when applied to an SIRS model with an appropriate combination of parameters and stochastic events. For such results to be credible, however, it is necessary that the parameter combinations that produce best-fitting simulations are biologically plausible, approximately consistent from one simulation to another, and are not easily affected by random events within the model. The model presented here fails the latter 2 conditions and therefore suggests that seasonal changes in vitamin D levels are not the predominant determinant of influenza seasonality in temperate regions.

The generalizability of our study may be limited by the fact that vitamin D levels were measured only in male health professionals; nonetheless, the mean 25(OH)D values were similar to those of a nationally representative study population [22]. Within the Health Professionals vitamin D dataset used here [18], no age-related differences in seasonal vitamin D levels were evident. In the future, should more detailed data representing a broader demography become available in which age-stratified vitamin D effects are evident, these effects could be incorporated and tested within the model framework.

Given that vitamin D affects the immune system [7], [8], one can hypothesize that severity and duration of influenza infection would also be modulated by vitamin D levels; however, we are unaware of any observational evidence supporting this hypothesis. Should such findings emerge in the future, this evidence would motivate proper testing of vitamin D-induced changes in the severity or duration of infection on the seasonality of influenza. This study was also limited by a lack of detailed *daily* 25(OH)D data; however, 25(OH)D levels are not subject to drastic day-to-day variations so this shortcoming likely did not affect our results.

Previous work indicates that increased solar radiation anomalies are associated with the onset of individual influenza outbreaks [6]. This association between increased sunlight availability and increased influenza transmission is incongruous with the vitamin D hypothesis (i.e. of the wrong sign) and also undermines the notion that vitamin D is a dominant driver of influenza transmission in temperate regions. While it remains possible that low levels of vitamin D could contribute to influenza occurrence, we conclude that present evidence for seasonal variation in serum vitamin D metabolite levels as a driver for influenza seasonality is considerably weaker than that for other proposed mechanisms, in particular seasonal variation in AH and seasonal changes in host aggregation driven by school terms.

## References

[1] Cauchemez S, Valleron AJ, Boelle PY, Flahault A, Ferguson NM (2008). Estimating the impact of school closure on influenza transmission
from Sentinel data.. Nature.

[2] Cauchemez S, Ferguson NM, Wachtel C, Tegnell A, Saour G (2009). Closure of schools during an influenza pandemic.. Lancet Inf Dis.

[3] Cannell JJ, Vieth R, Umhau JC, Holick MF, Grant WB (2006). Epidemic influenza and vitamin D.. Epidemiol Infect.

[4] Cannell JJ, Zasloff M, Garland CF, Scragg R, Giovannucci E (2008). On the epidemiology of influenza.. Virology Journal.

[5] Shaman J, Kohn MA (2009). Absolute Humidity Modulates Influenza Survival, Transmission and
Seasonality.. Proc Natl Acad Sci USA,.

[6] Shaman J, Pitzer VE, Viboud C, Grenfell BT, Lipsitch M (2010). Absolute Humidity and the Seasonal Onset of Influenza in the
Continental US.. PLoS Biology.

[7] Wang TT, Nestel FP, Bourdeau W, Nagal Y, Wang QY (2004). Cutting edge: 1,25-dihydroxyvitamin D3 is a direct inducer of
antimicrobial peptide gene expression.. J Immunol.

[8] Liu PT, Stenger S, Li HY, Wenzel L, Tan BH (2006). Toll-like receptor triggering of a vitamin D-mediated human
antimicrobial response.. Science.

[9] Yamshchikov AV, Desai NS, Blumberg HM, Ziegler TR, Tangpricha V (2009). Vitamin D for treatment and prevention of infectious diseases: a
systematic review of randomized controlled trials.. Endocr Pract.

[10] Urashima M, Segawa T, Okazaki M, Kurihara M, Wada Y (2010). Randomized trial of vitamin D supplementation to prevent seasonal
influenza A in schoolchildren.. Am J Clin Nutr.

[11] Sabetta JR, DePetrillo P, Cipriani RJ, Smardin J, Burns LA (2010). Serum 25-Hydroxyvitamin D and the Incidence of Acute Viral
Respiratory Tract Infections in Healthy Adults.. PLoS ONE.

[12] Young GA, Underdahl NR, Carpenter LE (1949). Vitamin-D intake and susceptibility of mice to experimental swine
influenza virus infection.. Proc Soc Exper Biol Med.

[13] Holmes AD, Pigott MG, Sawyer WA, Comstock L (1932). Vitamins aid reduction of lost time in industry.. Industrial and Engineering Chemistry.

[14] HopeSimpson RE (1981). The role of season in the epidemiology of
influenza.. J Hygiene.

[15] Laaksi I, Ruohola JP, Tuohimaa P, Auvinen A, Haataja R (2007). An association of serum vitamin D concentrations <40 nmol/L
with acute respiratory tract infection in young Finnish men.. Am J Clin Nutrition.

[16] Li-Ng M, Aloia JF, Pollack S, Cunha BA, Mikhail M (2009). A randomized controlled trial of vitamin D3 supplementation for
the prevention of symptomatic upper respiratory tract
infections.. Epidemiology and Infection.

[17] Ginde AA, Mansbach JM, Camargo CA (2009). Association between serum 25-hydroxyvitamin D level and upper
respiratory tract infection in the Third National Health and Nutrition
Examination Survey.. Archives Internal Med.

[18] Giovannucci E, Liu Y, Rimm EB, Hollis BW, Fuchs CS (2006). Prospective study of predictors of vitamin D status and cancer
incidence and mortality in men.. J Natl Cancer Inst.

[19] Lips P, Duong T, Oleksik A, Black D, Cumming S (2001). A global study of vitamin D status and parathyroid function in
postmenopausal women with osteoporosis: baseline data from the multiple
outcomes of raloxifene evaluation clinical trial.. J Clin Endocrinol Metab.

[20] Simonsen L, Reichert TA, Viboud C, Blackwelder WC, Taylor RJ (2005). Impact of influenza vaccination on seasonal mortality in the US
elderly population.. Arch Intern Med.

[21] Viboud C, Bjornstad ON, Smith DL, Simonsen L, Miller MA (2006). Synchrony, waves, and spatial hierarchies in the spread of
influenza.. Science.

[22] Martins D, Wolf M, Pan D, Zadshir A, Tareen N (2007). Prevalence of cardiovascular risk factors and the serum levels of
25-hydroxyvitamin D in the United States: data from the Third National
Health and Nutrition Examination Survey.. Arch Intern Med.

